# Artificial Intelligence in Cardiovascular Imaging: Current Landscape, Clinical Impact, and Future Directions

**DOI:** 10.15190/d.2025.10

**Published:** 2025-06-30

**Authors:** Sudeep Edpuganti, Amna Shamim, Vilina Hemant Gangolli, Ranasinghe Arachchige Dona Kashmira Nawodi Weerasekara, Amulya Yellamilli

**Affiliations:** ^1^Department of Medicine, Faculty of Medicine, Tbilisi State Medical University, Tbilisi, Georgia; ^2^Department of Medicine, School of Health Sciences, The University of Georgia, Tbilisi, Georgia

**Keywords:** Artificial Intelligence; Cardiovascular Imaging; Deep Learning; Diagnostic Accuracy; Regulatory Challenges.

## Abstract

Cardiovascular (CV) imaging is rapidly transforming with the advent of artificial intelligence (AI), automating and augmenting diagnostic pipelines in echocardiography, computed tomography (CT), magnetic resonance imaging (MRI), and nuclear imaging. In this review, we summarize recent developments in convolutional neural networks for real-time echocardiographic interpretation, deep learning for coronary artery calcium scoring that achieves near-perfect agreement with manual methods, and AI-driven plaque quantification and stenosis detection on coronary CT angiography, which achieves an accuracy of ≥ 96%. FDA-approved platforms (e.g., Aidoc, HeartFlow, Caption Health) emphasize clinical translation, while automated segmentation and perfusion analysis in cardiac MRI produce Dice coefficients ≥ 0.93. We critically analyze persistent issues, algorithmic bias, explainability, data privacy, regulatory heterogeneity, and medico-legal liability. We also discuss risk-reduction tactics, such as federated learning and human-in-the-loop oversight. Reactive diagnostics will allow proactive, personalized treatment in the future, assuming we look ahead, thanks to multimodal AI, wearable sensors, and predictive analytics. For AI to fully optimize cardiovascular care, thorough validation, open algorithmic design, and interdisciplinary cooperation will be necessary.

## Summary

1. Introduction

2. Overview of Artificial Intelligence in Imaging

3. AI in Echocardiography

4. AI in cardiac CT and coronary imaging

5. AI in cardiac MRI and nuclear imaging

6. Clinical impact and Diagnostic accuracy

7. Challenges, Limitations, and Ethics

8. Future directions

9. Conclusion

## 1. Introduction

Cardiovascular diseases (CVDs) refer to the illnesses that affect the heart and blood vessels and are the leading cause of death globally. Cardiovascular diseases (CVDs) were the leading cause of death in 2019, with an impact mainly seen in low and middle-income countries according to the World Health Organization^1^.

In 2019 alone, they were responsible for an estimated 17.9 million deaths, of which over four-fifths are due to Heart attack and stroke^1^. Along with mortality, CVD is also a major contributor to economic burden, strain on the healthcare system, and disability-adjusted life years (DALYs). According to the Global Burden of Disease (GBD) 2019 study, CVDs have accounted for more than 381 million DALYs worldwide, ischemic heart disease and stroke being the top two with 182 million and 143 million^2^.

Precise diagnosis and risk assessment are needed for the management of CVDs. There are many non-invasive imaging techniques, such as Echocardiography, Cardiac Computed Tomography (Cardiac CT), Cardiac Magnetic Resonance Imaging (CMR or Cardiac MRI), Nuclear Imaging, all of which have become essential in diagnosing CVDs. Echocardiography is most commonly used for diagnosing conditions like heart failure (HF) by determining the left ventricular ejection fraction (LVEF)^3^. It is also used for detecting structural abnormalities^4^ and regional wall motion abnormalities^3^. Cardiac CT and coronary artery calcium (CAC) scoring obtained with a breath hold of roughly 3 to 5 seconds gives a non-invasive and non-contrast evaluation of atherosclerosis, playing a vital role in early detection and preventive cardiology^5^. Cardiac Magnetic Resonance Imaging (CMR) has become a gold standard for myocardial tissue characterization, allowing for the highly precise detection of ischemia, fibrosis, and viability^6^. Nuclear imaging methods like PET and SPECT play an important role in evaluating the myocardial perfusion and viability, particularly in patients with already diagnosed or suspected coronary artery disease (CAD)^7^.

Although cardiovascular imaging is quite important in clinical practice, traditional imaging techniques have limitations that compromise diagnostic accuracy and clinical decision-making. Echocardiography, despite being widely used, is highly operator-dependent and shows interobserver variability, particularly in LVEF assessment^8^. Post-scan processing and manual analysis of imaging datasets, particularly in cardiac MRI, remain time-consuming processes that can delay the diagnosis^6^.

In a high-volume clinical setting such as emergency departments, reliance on manual interpretation of the imaging often contributes to delays in diagnosis and workflow bottlenecks^9^. These ongoing difficulties with conventional imaging draw attention to the need for technological innovations like artificial intelligence to improve diagnostic speed, standardization.

Given the growing importance of Artificial Intelligence (AI) across cardiovascular imaging Modalities, a comprehensive review is needed not only of its current clinical applications but also of performance comparisons, FDA-approved technologies, and future directions. This review aims to synthesize recent developments and address the challenges associated with real-world implementation.

## 2. Overview of artificial intelligence in imaging

Artificial Intelligence refers to algorithmic systems capable of autonomous pattern recognition and decision-making based on complex, multidimensional datasets. This usually entails using data such as medical records or information taken from photos to determine the best course of treatment, detect a new condition, or anticipate a likely diagnosis^10^. Recent advances in deep neural architectures, expanding biomedical datasets, and accessible computational frameworks have catalyzed AI’s integration into clinical imaging^11^.

Machine learning (ML), from classical regressions and support vector machines to convolutional neural networks (CNNs), underpins most modern AI tools in imaging. A branch of ML called deep learning (DL) makes use of deep neural networks^12,13^.

### 2.1 Biobanks and Bioresources

Big data includes genetic information, imaging data, medical health records, patient outcomes, and outcome data. Structured big data gathering includes “biobanks” and “bio-resources”^14^. An additional type is an atlas, which combines data to offer reference information on structural variation, like that found in the heart^15^. AI models perform better with larger data sets. To be appropriate for ML, many datasets must be restructured into features, which are discrete informative units like motion vectors, clinical reports, or imaging metrics like pixel brightness^16^. The effectiveness of computational methods in producing an AI will depend on the caliber, precision, and depth of features in the data. When improper or misclassified data is provided, it indicates that the dataset does not sufficiently reflect the real world for ML to produce a model^10^.

### 2.2 Computational approaches

ML algorithms can be trained in two broad ways: supervised learning and unsupervised learning. Another method that utilises many models to increase forecast accuracy and robustness is ensemble learning.

#### Supervised learning

In supervised learning, the model iteratively evaluates the data with each feature being chosen, processed, and weighted to determine the optimal combination that yields the intended result. For many years, regression analysis has been one of the first and most basic types of supervised learning and has been applied extensively. More advanced methods for carrying out intricate associative analyses, such as random forests and support vector machines, have surfaced in recent years^17,18^.

It is essential to assess a supervised learning system's performance on unseen data once it has been taught. The dataset is usually divided into distinct subsets for algorithm testing and training. Sometimes, a different subset of the training data is reserved for algorithm optimization. Testing must be done using data that isn't used for training^13^. How well an algorithm generalises to new, unknown data depends on two important factors^10^; how well the algorithm has been tuned to prevent overfitting, a condition in which the model captures noise or random oscillations in the training data, resulting in subpar performance on new data, and^11^ how representative the sampled dataset is of the larger target population^13^.

#### Unsupervised learning

Unsupervised learning aims to find innate, recurring patterns in the data itself, rather than fitting data to a preset result. Principal component analysis (PCA) and cluster analysis, including “k-means” clustering, are among the methods used in this approach^18^. The researcher's domain knowledge can add a degree of subjectivity to the clustering method selection process, which is frequently impacted by the data and intended applications as well. More recently, sophisticated clustering techniques have been created to more successfully find patient similarities.

AI's ability to learn and distinguish is greatly enhanced by combining several statistical techniques. For instance, patients with known outcomes can be subjected to unsupervised learning approaches in order to find distinctive characteristics that could subsequently be included in supervised models^19^.

Unsupervised learning has a lot of promise, although it is still less frequently used in medical applications than supervised learning. This is especially helpful in the medical field, where it can be costly and time-consuming to manually annotate big datasets for supervised learning. Among the most popular unsupervised machine learning algorithms are principal component analysis and clustering techniques like “k-means” or hierarchical clustering^13^. As unsupervised learning develops further, its incorporation into healthcare processes requires cautious implementation and validation of its results.

### 2.3 Implementation and verification

Assessing whether an ML model retains accuracy when exposed to fresh data is essential after it has been developed. To prevent bias, this entails utilising different datasets for training and testing^20,21^. It has been demonstrated that proper validation uses methods such as “x-fold cross-validation”, often known as 10-fold, which iteratively tests the model on unseen data and yields more dependable findings than straightforward split-sample approaches ^20,21^. Determining the model's resilience and generalisability limits can be aided by adding more datasets with minor modifications. Before being approved by regulators, AI applications, including automated measures, clinical alarm systems, and diagnostic support tools, must pass a rigorous review to guarantee dependability and safety^10^.

### 2.4 AI application for Interpretation 

#### Cluster Analysis and disease phenotyping 

Cluster analysis is an unsupervised machine learning methodology that offers a method for generating homogenous, linked groupings from hidden patterns in data. In order to identify pertinent illness phenotypes and taxonomies, clustering can be a very useful technique for comprehending the connections between clinical data from electronic medical records and clinical imaging. Although clustering has become increasingly popular in many other domains during the past few decades, its use in cardiology has remained rather limited^22^.

Clustering can be used as a pre-processing tool for other ML algorithms, as an exploratory or hypothesis development and testing method, or as a standalone tool to gain understanding of the distribution and intricate synopsis of patient data or cardiac imaging parameters^10^. For very heterogeneous cardiac disorders, hierarchical clustering is especially pertinent. One prominent instance is its application to diabetes and heart failure with preserved ejection fraction (HFpEF), where clustering of clinical and echocardiographic data has identified unique phenotypes, enhanced risk assessment and directing individualised care. Building on these fundamental approaches, various artificial intelligence tools are currently being used more and more to improve the analysis and interpretation of cardiac imaging.

### 2.5 Artificial Intelligence Techniques for Cardiac Imaging

Two main methods for using AI in cardiac imaging have been documented. In order to predict diagnostic or prognostic outcomes, the first method applies classical machine learning (ML) techniques to huge datasets made up of clinical and or pre-computed imaging information^13^. The second, more sophisticated method makes use of DL techniques, where AI models analyse real images directly to carry out tasks like outcome prediction or image segmentation. This eliminates the need for traditional “feature engineering”, which involves computation and extraction of "custom-tailored" imaging variables.

#### Classical AI

The fundamental idea behind classical AI in cardiac imaging is that a collection of weak base classifiers can be combined into a strong, accurate model by automatically adjusting their weightings. New sets of base classifier predictions are produced at each iteration, along with updated weighting distributions. The overall classifier, the ML risk score, which ranges from 0 to 1, is then obtained as a continuous estimate of the projected risk by combining these predictions using weighted majority voting. Models were created using LogitBoost with 10-fold cross-validation, and features were automatically chosen using information gain ranking. In comparison to existing risk indices, the resulting ML risk score, which combined clinical and imaging data, achieved a much higher performance^20^.

Similarly, AI outperformed coronary calcium scoring (CCS) in the MESA (Multi-Ethnic Study of Atherosclerosis) cohort, which included approximately 6,800 asymptomatic people, in predicting adverse cardiovascular events^23^. Moreover, AI was also utilised to forecast ischaemia specific to lesions. When compared to stenosis alone, an evaluation of plaque characteristics was found to improve the discrimination of lesion-specific ischaemia in the trial^24^. 

#### Deep learning

In cardiac imaging, deep learning (DL) has yielded advances in diagnosis, prediction, interpretation, and image enhancement, such as anatomical labelling and improved image quality^25^. DL applications in medical imaging have grown quickly after their success in computer vision, especially in automated tasks such as organ and lesion segmentation, detection, and, to a lesser extent, classification. With consistent superiority over conventional feature-engineered techniques and matching expert reader variability, DL has been successfully used in cardiology for coronary centerline extraction, cardiac magnetic resonance (CMR) segmentation, left ventricular (LV) CT and ultrasound imaging, and coronary calcium scoring (CCS)^25^.

## 3. AI in Echocardiography

The most used imaging technique in cardiology is echocardiography, which is becoming more and more popular among non-cardiologists. However, thorough operator training is necessary for reliable echocardiographic data interpretation. With automated, high-quality computer-optimized picture acquisition, automated measurements, and algorithms for quick and accurate evaluation of heart function, AI has started to improve echocardiography in real-world clinical situations significantly^26^. AI has the potential to greatly increase the effectiveness and precision of echocardiogram assessments by lowering human variability^27^.

The following section explores how artificial intelligence is improving diagnostic accuracy and efficiency in echocardiography by revolutionising picture acquisition and optimisation.

### 3.1 Acquisition and Optimisation of Images

#### Segmentation 

Classifying standardised transthoracic echocardiographic images is the next crucial step in echocardiographic interpretation. Madani and colleagues^28^ developed a vendor-agnostic DL model trained on 247 real-world echocardiograms, comprising approximately 200,000 clinically acquired images. Surprisingly, their model outperformed board-certified echocardiographers in the identical experiment, classifying 15 important transthoracic echocardiographic images with 98% accuracy^26^. The next development, which builds on precise segmentation, is automated quantification, simplifying important measurements and improving clinical evaluation consistency.

#### Automated Measurements

Several suppliers presently use commercially available automated measurement procedures for 2-dimensional (2D) and 3-dimensional (3D) echocardiographic datasets. These tools minimise human mistakes and reduce operator dependency, which improves reproducibility. The manual tracing of endocardial boundaries is a laborious and subjective procedure that is primarily reliant on the operator's experience when evaluating the EF using 2D echocardiography^29^.

AI is included in the process to increase its efficiency. It is now simpler to assess cardiac chamber dimensions, volumes, stroke volume, EF, and wall thickness by employing automated border-detection algorithms and utilising ECG data to detect end-systolic and end-diastolic frames. Near real-time evaluations with little manual correction are made possible by machine learning-assisted 3D echocardiography, which facilitates effective investigation of left and right ventricular function. Automatic cine-derived LVEF correlates with conventional volume-derived EF at 90% or above, according to studies^26^.

The American Society of Echocardiography has suggested increasing the use of 3D echocardiography to evaluate ventricular cavity volumes in light of these developments^26^. CMR, frequently unavailable in many healthcare settings outside of major tertiary centres, could be significantly reduced by integrating AI with 3D echocardiography into everyday clinical practice. Advanced imaging techniques have opened the door for dynamic cardiac modelling, which provides deeper insights into the anatomy and function of the heart beyond routine data.

#### Cardiac Modelling

For cardiac evaluation, M-mode and Doppler echocardiography are still frequently utilised techniques. M-mode offers great temporal and spatial resolution to catch minor motion patterns with a single interrogation beam. In contrast, Doppler echocardiography provides velocity-time pictures that are utilised to evaluate problems such as valve regurgitation and stenosis.

M-mode parameters like right ventricular internal diameter in diastole, interventricular septal thickness, left ventricular (LV) internal diameter, and posterior wall thickness during both systole and diastole, as well as several important Doppler-derived measurements like mitral inflow, aortic, and tricuspid regurgitation, can be automated by ML^30^. In AI-driven research, B-mode echocardiography has also been utilised to identify and measure aberrant regional LV wall motion, which helps early CHD detection.

The creation of end-to-end AI models that immediately analyse echocardiograms to draw diagnostic findings, eschewing the need for intermediate manual procedures, is an emerging trend. Moreover, an increasing amount of research is being done on developing customised cardiac models that employ patient data to mimic heart shape and function, electrophysiology, biomechanics, and haemodynamics^31-34^. With their thorough understanding of cardiovascular health and disease, these AI-driven methods have enormous potential to advance precision medicine^35,36^. AI has transformed cardiac deformation analysis in tandem with advances in structural and functional modelling, especially through the automated evaluation of GLS.

#### Global Longitudinal Strain

The fully automated measurement of 2D left ventricular (LV) global longitudinal strain (GLS) is a commonly used application of artificial intelligence in echocardiography^37^. AI facilitates automatic myocardial motion monitoring, the quick recognition and categorisation of standard echocardiographic images, and the assessment of GLS, especially in patients suffering from heart failure or acute myocardial infarction. With high feasibility (98%) and exceptional precision, these AI-powered devices can evaluate EF and GLS simultaneously in around 8 seconds. AI is being used more and more to identify and distinguish abnormal patterns in addition to measuring heart function, improving the accuracy of diagnosis for a variety of CVDs.

#### Disease Detection

Artificial intelligence has shown great promise in recognising disease-specific echocardiogram patterns with precision on par with that of trained medical professionals. To identify characteristics of diseases, including HCM, cardiac amyloidosis, and pulmonary hypertension, algorithms have been devised^38^.

#### Mitral Regurgitation

A machine learning model was used to evaluate mitral valve regurgitation in 139 people in a study by Moghaddasi and Nourian, 139 people participated in the study, comprising 37 healthy controls from the Tehran Heart Centre and 102 patients with mild, moderate, and severe forms of mitral regurgitation (34, 32, and 36)^39^. The algorithm correctly identified normal valves (99.5%) and classified regurgitation as mild (99.38%), moderate (99.31%), and severe (99.59%). 99.38% and 99.63%, respectively, were the overall sensitivity and specificity. Additionally, for all grades of mitral regurgitation, a support vector machine model showed over 99% accuracy.

#### Aortic Stenosis

To evaluate severe high-gradient aortic stenosis, Playford et al.^40^ compared an AI-based approach with the conventional continuity equation. The AI model showed a sensitivity and specificity of 91.4% at a probability cutoff of 0.065425, identifying 2,382 patients (7.38%) with severe aortic stenosis in a large test cohort of 32,574 people. While traditional methods rely on left ventricular Outflow tract (LVOT) size and velocity, the AI model used phenotypic features and correctly assessed stenosis severity in 95.3% of patients, compared to 73.9% accuracy with the continuity equation. It remained a reliable predictor of long-term mortality after adjusting for transaortic gradients and stroke volume index.

#### Other Cardiac Conditions

AI has also shown promise in the diagnosis and categorisation of a number of additional cardiac disorders. Using segmental deformation and strain rate curves, it has diagnosed MI with 87% accuracy and used multiple classifiers to differentiate between athlete's heart and hypertrophic cardiomyopathy. With an Area under the curve (AUC) of 0.96, an associative memory classifier trained on speckle tracking echocardiography (STE) was able to distinguish RCM from CP^10^.

AI's incorporation into the echocardiographic workflow can revolutionise not only interpretation but the complete imaging process, from acquisition to reporting, as it continues to improve diagnosis accuracy across a range of cardiac diseases.

#### Workflow for Echocardiography

It is anticipated that the workflow for echocardiogram interpretation will be drastically changed by the addition of AI. Currently, a physician manually examines the images to evaluate wall motion, cardiac function, and valvular anomalies, utilising 2D, 3D, and Doppler modalities after a sonographer acquires the images, which cover parasternal, apical, subcostal, suprasternal, off-axis, and Doppler modalities.

By automatically categorising and arranging images according to their therapeutic significance, AI can expedite this procedure. For example, the system might show all pertinent LV cine images if the clinical question concerns LV wall motion. It may display 2D or 3D LVOT images in conjunction with Doppler tracings of peak transvalvular velocities and stroke volume in cases of aortic stenosis. This query-driven structure improves efficiency and clarity. Additionally, in order to provide thorough evaluations of the severity of stenosis, AI can incorporate metrics such as GLS and stroke volume index from 3D data^41,42^.

Automating longitudinal comparisons is another useful application. AI can improve speed and accuracy by retrieving and aligning corresponding images from earlier studies for direct side-by-side assessment, removing the need for previous reports. Accreditation standards that emphasise image-based evaluations are supported by this^43^.

However, AI may also completely change how echocardiography interpretation is prioritised. At the moment, there are several categories for urgency, such as "stat," "ICU," and "routine." By automatically recognising and alerting key findings like cardiogenic shock, huge pericardial effusions, or severe right ventricular dysfunction for prompt clinician assessment, AI systems may potentially improve triaging. By selecting high-risk cases and enabling quicker action, AI-enabled real-time triage delivers substantial clinical value in emergency and intensive care unit settings where time-sensitive judgements are essential. By guaranteeing that patients in life-threatening circumstances receive timely care, this integration may enhance acute care settings' resource allocation and results. Table 1 summarises the valvular and structural heart disease diagnosis accuracy, sensitivity, and specificity of AI models.

**Table 1:** AI Performance Metrics in Echocardiographic Disease Detection

Condition	AI Method/Model	Sample Population	Metric type	Performance	Ref
MR	ML Model	139 (102 MR patients + 37 normal)	Normal valve detection	99.5% accuracy	^40^
		139	Mild regurgitation	99.38% accuracy	^40^
		139	Moderate regurgitation	99.31% accuracy	^40^
		139	Severe regurgitation	99.59% accuracy	^40^
		139	Overall Sensitivity	99.38% accuracy	^40^
		139	Overall specificity	99.63% accuracy	^40^
	Support Vector Machine	Not specified	All grades classification	>99% accuracy	^10^
Aortic Stenosis	Phenotype-based AI model	32,574 total; 2,382 with severe AS	Severity classification	95.3% accuracy	^41^
	Continuity equation(traditional)	32,574 total; 2,382 with severe AS	Severity classification	73.9% accuracy	^41^
	Phenotype-based AI model	32,574 total; 2,382 with severe AS	Prognosis	Reliable predictor	^41^
MI	Segmental deformation & Strain rate	Not specified	Diagnostic Performance	87% accuracy	^10^
Athlete’s heart vs HCM	Multiple classifiers	Not specified	Diagnostic discrimination ability	Not specified	^10^
RCM vs CP	Associative memory classifier + STE	Not specified	Discrimination accuracy (AUC)	0.96	^10^

## 4. AI in cardiac CT and coronary imaging

### 4.1 Role of CT in Cardiology

Across all areas of cardiovascular medicine, Cardiac Computed Tomography (CCT) plays an essential and crucial role in clinical decision making^44^. From evaluation of chest pain to assessment of structural procedures, the range of indications for CCT has become wide. This is primarily due to the evolution of the CT scanner technology, which has led to sharper and detailed visualization of coronary vessels and other cardiovascular structures^45^.

Furthermore, CCT is also used in pediatric populations, such as newborns. It is used to evaluate complicated cardiovascular anatomy, which is difficult to visualize using echocardiography or other imaging modalities. It can also be used as an adjunct to echocardiography and cardiac catheterization when additional anatomical information is required before planning and/or assessing the feasibility of surgery^46^.

Another non-invasive imaging technique is Coronary Computed Tomography Angiography (CCTA), which is an alternative to the invasive coronary angiography (ICA) and is extremely helpful in determining the extent of coronary artery stenosis. In all patients who are likely to have CAD, it has now become the first-line diagnostic imaging test^47^. An important advantage of CCTA is that it can characterize coronary lesions into obstructive or non-obstructive. Additionally, it can also distinguish atherosclerotic plaques as calcified, noncalcified, and high-risk^45^.

Moreover, for structural heart disease interventions, for example, transcatheter aortic valve replacement, CCTA plays a significant role in the selection of the site for vascular access. It can provide information regarding radial and iliofemoral anatomy by increasing scanning range from the upper extremities to the pelvic area^48^.

Besides CCTA, another application of CT is Fractional Flow Reserve computed tomography (FFR CT) and Myocardial CT perfusion (CTP). FFR CT is a new non-invasive imaging method used for the assessment of the plaque in CAD. Whereas, Myocardial CTP assesses myocardial perfusion during rest and stress to check for ischemic areas^49^.

### 4.2 Applications of AI in CT

#### Automated CAC Score

Due to the advancements in imaging technology and AI techniques, such as deep learning (DL), AI has been incorporated into all imaging modalities^50^. DL is primarily characterized by convolutional neural networks (CNNs). The role of CNNs in the field of cardiac imaging is expanding. One of its applications is the automated detection and quantification of coronary calcification, resulting in an accurate and efficient coronary artery calcium (CAC) score^51^.

CAC score, also referred to as the Agatston score, is a marker for atherosclerosis and is the most frequently used method for assessing the CAD burden in patients. In addition, it is an important prognostic factor and predictor of adverse cardiovascular events. Electrocardiogram (ECG)-gated non-contrast enhanced cardiac computed tomography (CT) and non-gated chest CT are the two imaging techniques used for the detection of CAC^52^.

A study by Abdelrahman et al.^50^ on 79 CT scans using the CNN DL model revealed that the model has almost perfect results and is much faster when compared to manual CAC scoring for gated cardiac CT(κ= 0.89). In addition, the same article discussed another study done using the CNN model on 1811 cases, which had high sensitivity (99%) and specificity (100%) when applied to a larger population. Similarly, for non-gated chest CT, the same article also discussed another CT study using 168 patients; the automated CAC scores agreed with the manual scoring (Pearson’s correlation coefficient = 0.921) ^50^.

Although non-gated chest CTs have the advantage of measuring CAC simultaneously while the patients undergo the scan for other purposes, without further radiation exposure, vessel-specific CAC is difficult to assess without ECG synchronization^53^.

Besides CAC scoring, AI has expanded into advanced plaque characterization and quantification.

#### Plaque Quantification

In the past, the most important factor that influenced the treatment options for atherosclerosis was the extent of stenosis of the artery. But over the last few years, many studies have shown that morphology and composition of the plaque also play a crucial role in plaque vulnerability or stability and determine the risk of rupture, occlusion, and embolus formation. Routine CTA offers qualitative, semi-quantitative, and quantitative evaluation of atherosclerotic plaque vulnerability with its characteristic features^54^.

For the enhancement of automated quantification of plaque, numerous AI algorithms have been developed. A study by Chen et al.^55^ discussed another study, which was carried out using 894 CCTA scans of 308 patients, and it showed good agreement with expert analysis to quantify total (ICC=0.88), Non-calcified (ICC=0.84), and calcified (ICC=0.99) plaque volumes, which were achieved with the DL model. Furthermore, it also exhibited outstanding agreement with intravascular ultrasound (IVUS) for the assessment of total plaque volume, along with reduced analysis time compared to manual analysis. However, these AI models require some preprocessing procedures and hence, are not completely automated. Novel AI algorithms are being developed to get rid of the manual processes^55^. Another application of AI is the improvement of image quality and the image noise.

#### CCTA image enhancement and noise reduction

Due to the widespread use of CCTA, the exposure to ionizing radiation has substantially increased. Several advancements have been made to reduce the radiation dose, but decreasing the dose further increases image noise, which causes image quality degradation. To tackle this problem, image reconstruction algorithms, for example, iterative reconstruction, which is a commonly used noise reduction technique, are being used along with the DL model^56,57^. In a study done using 82 patients, a dataset of 164 CT scan images was created (82 original images and 82 denoised images); the inter-observer agreement for subjective image quality showed good agreement (Cohen's Kappa, 0.619; ICC, 0.751). In addition, for objective sharpness, the denoised images had a lesser average ERD (edge rise distance) than the original images, which signifies that the denoised images were sharper (original vs denoised images: 0.98 ± 0.08 vs. 0.90 ± 0.08). It also improves the subjective and objective qualities of the image without over-smoothing^56^. Furthermore, CCTA can also detect the degree of coronary artery stenosis in a short amount of time. 

#### Stenosis Detection

On CCTA, the diagnosis and staging of coronary artery stenosis requires substantial experience, is prone to errors, and is time-consuming^58,59^. To help simplify the process of stenosis detection, AI algorithms have proven to be helpful for the detection of varying degrees of coronary stenosis and calcified plaque^60^. Automatic interpretation of the scan can be performed using a deep neural network, an AI technique. A deep learning model (DLM) was able to detect if the coronary arteries were obstructed, nonobstructed, or normal, similar to trained physicians. In a study performed using 53 patients, the model performed optimally, with an accuracy of 96% for distinguishing between patients with or without ≥50% stenosis^58^. In addition to DLM, ML models have also been developed to identify CCTA images for coronary stenosis^61^.In addition to stenosis detection, CCTA can also help evaluate the cardiovascular risks of individuals in a more personalized manner.

#### Risk Stratification

Cardiovascular diseases (CVD), one of the leading causes of death worldwide, require early assessment of risks^62^. Framingham Risk Score (FRS) and the Pooled Cohort Equations (PCE) and other conventional risk assessment models mainly rely on demographic and clinical indicators consisting of age, sex, blood pressure levels, cholesterol levels, smoking habits, and diabetes, which may result in underestimation or overestimation of the risk^63^. Hence, in cardiovascular imaging, AI models such as ML and DL are being used for risk stratification^61^.

As the application of CCTA for the detection of CAD is increasing, this provides us with the opportunity to use data available from CCTA, for example, disease activity and coronary atherosclerosis, to define the cardiovascular risk. A novel AI-based risk evaluation model has been developed called the AI-Risk model. It combines the Fat Attenuation Index (FAI), degree of coronary atherosclerosis, along with the conventional risk factors of the individual. This offers a personalized assessment of risk and thus helps in the early initiation of preventive treatment^64,65^. A study performed on 3393 patients demonstrated that the AI-Risk Model was able to strongly predict major adverse cardiac events (MACE) and cardiac mortality over 10 years, both in the presence and absence of coronary atherosclerosis^65^. The comprehensive scores are explicitly presented in Table 2 below.

Another commonly performed imaging test, abdominal CT, can potentially be used to predict cardiac risks. This can be accomplished with the help of the Deep Learning-based Cardiovascular Disease Incident (DL-CVDi) score. The DL-CVDi score can detect complicated, non-linear relationships between anatomical features and CVD risk. Increased risk of CVD incidents was observed in individuals with high DL-CVDi scores. However, further research should be done to confirm these findings^63^.

Although AI has the potential to dramatically change the field of cardiovascular medicine and imaging, some concerns do exist regarding its application. Concerns range from data security, trust, transparency, and risk of harm to the patient. Additionally, if the AI is trained on a small dataset, it may result in bias^66^.**Table 2** outlines the performance of AI models applied in CT and coronary imaging.

**Table 2:** Performance of AI Models in CT-Based Coronary Imaging: CAC Scoring, Plaque Analysis, Image Enhancement, and Risk Stratification

Application	AI Method/Model	Study population	Metric type	Performance	Ref
CAC score (ECG gated)	DL model	Population not specified	Cohen’s Kappa (κ)	0.89	^50^
		1811	Sensitivity	99%	^50^
		1811	Specificity	100%	^50^
CAC score (Non-ECG gated)	DL model	168	Pearson’s correlation coefficient	0.921	^50^
Plaque quantification	DL Method	308	Inter-class Correlation (ICC)	Total (ICC=0.88) Noncalcified (ICC=0.84) Calcified (ICC=0.99)	^55^
Subjective Image Quality	DL Model	82	Cohen’s Kappa (κ), ICC	κ = 0.619 ICC = 0.751	^56^
Objective Sharpness of Denoised Image	DL Model	82	Average ERD (Edge Rise Distance)	Original image = 0.98 ± 0.08; Denoised image = 0.90 ± 0.08	^56^
Stenosis detection	DL Model	53	Accuracy	96%	^58^
Risk Stratification (Cardiac Mortality)	AI-Risk Model	3393	AUC score	Whole population = 0.854; Without Obstructive CAD = 0.816; With obstructive CAD = 0.773	^65^
Risk Stratification (MACE)	AI-Risk Model	3393	AUC score	Whole population = 0.805; Without Obstructive CAD = 0.748; With obstructive CAD = 0.764	^65^

### 4.3 Performance benchmarks - AUC scores, Dice coefficients

Several AI models, such as DL and ML, are being developed to aid in identifying coronary artery stenosis on CCTA images. For detection of obstructive CHD, the different ML models, the neural network with multi-task learning, demonstrated a better predictive power to identify ≥70% coronary artery stenosis when compared to other ML models for example, Random Forest (AUC 0.695), XGBoost (AUC 0.732), and logistic regression (AUC 0.749). Compared to the traditional DL methods, the neural network-based model performed better, as confirmed by the findings. Furthermore, for the estimation of obstructive CHD prediction, when compared with PCE (AUC 0.719), CAD consortium score (AUC 0.696), and UDF scores (AUC 0.705), the neural network model possessed a greater area under the ROC curve^67^.

The prognosis of patients with CAD after PCI depends on the condition of the stent (if it is deformed or fractured) and ISR. CCTA helps with post-operative stent follow-up. Automated stent identification can be done using AI-based CCTA, which has a Dice similarity coefficient (DSC) of 0.96, for automated coronary stent segmentation, comparable to cardiologists^68^.

## 5. AI in cardiac MRI and Nuclear imaging

### 5.1 Introduction of AI in cardiac MRI

Cardiac magnetic resonance (CMR) is used to evaluate various heart conditions such as valvular diseases and cardiomyopathies. It is highly beneficial for estimating the heart’s EF, myocardial thickness, and scar patterns. The use of special settings, such as T1 and T2-weighted scans, aids in making an accurate diagnosis. Additional diagnostic procedures, such as LGE, play a crucial role in myocardium characterization as it is highly precise and sensitive to lesions of the myocardium, such as fibrosis and ischemia. This enables CMR to perform with higher accuracy at diagnosing pathologies compared to echocardiography. However, it has its limitations, such as being highly time-consuming and repeated breath-holding^69,70^.

By the implementation of AI in diagnostic procedures, the time taken for analyzing CMR is greatly reduced. This is done as ML, DL, and CNN-based AI models are trained on large data samples where they can analyze patterns and learn the relationship among them and apply them to newly obtained data autonomously^71^.

#### Advantages of AI in CMR

MRI images can be reconstructed through AI approaches such as k-space agonistic and k-space aware models. K-space-agonist models use a U-net CNN, which is trained to convert undersampled k-space data to corresponding images created from fully sampled k-space data. K-space-aware models apply the previous apprehension of the acquisition process with CNN’s aid. This approach is able to decrease the time taken for CMR as AI can generate high-quality images and has the competence to enhance the resolution of images from partial k-space data. AI has high denoising potential with a decreased amount of blurring. It can amplify Signal to Noise Ratio (SNR) and is useful in LGE where there is sparse SNR. AI with CNN architecture can be trained to predict tissue parameters across different weights past many heartbeats, which reduces scan time as the requirement of multiple images and time taken for recovery between images is no longer needed^72^.

#### CMR segmentation and perfusion analysis by AI

Assadi et al have trained an AI based on the information collected from 814 study participants. The study participants were not limited to one medical centre, and MRI machines from different manufacturers were used to train AI to reduce bias. The developed AI model was fully automated and can perform segmentation of heart chambers. This was able to estimate heart parameters such as end-systolic volume, end-diastolic volume, stroke volume, and EF in milliliters (mL) of all the chambers. This model has achieved a Spearman coefficient (ρ) for left heart parameters ranging from 0.98-0.91. ρ for right heart parameters range from 0.98-0.77. These results indicate a strong correlation between AI-generated and manual segmentations for the chamber^73^. Another CNN-based AI model developed by Xue et al has been trained on data obtained from 1034 patients. It was tested on 200 scans obtained from 105 patients, achieving a mean Dice ratio of 0.93 ± 0.04 and 0.93 ± 0.03 across different magnetic field strengths (3T and 1.5T, respectively) for myocardium segmentation. A P value of .93 and .84 was recorded at 3T for mean stress flow and mean rest flow, respectively. For 1.5T, the P value demonstrated is .97 and .93 for mean stress flow and mean rest flow, respectively^74^. A dice ratio value closer to 1 indicates more similarity, and a P value closer to 1 indicates similar performance, which indicates a high correlation between AI-based and manual performance.

#### Role of AI in myocardium characterization

Schwab et al developed a DL-based algorithm that performs faster segmentation of MI and microvascular obstruction (MVO) on LGE CMR. The AI was trained on data obtained from 142 study participants, which includes 144 LGE CMR images. The algorithm’s performance was further investigated on an independent set of 152 LGE CMR images, which consist of 121 ST-segment elevations and 129 without MVO. This model has achieved a mean DICE coefficient of 64.11% for infarct segmentation and 82.20% for MVO segmentation. This indicates that the developed AI was able to strongly recognize MVO regions and moderate detection of infarcts^75^.

### 5.2 Introduction of AI in Cardiac Nuclear Imaging

The application of AI in nuclear imaging of the heart has shown improved diagnostic accuracy and speed, as demonstrated in CMR. Myocardial perfusion imaging (MPI) procedures, such as SPECT and positron emission tomography (PET), are considered highly accurate non-invasive imaging modalities for cardiac pathologies, with PET being more sensitive. Hybrid imaging techniques further aid in improving diagnostic accuracy^76^. Application of certain AI models (DL and ML-based) is highly accurate in diagnosing CADs and ischemia, along with CNN’s denoising nature, which enhances the image. This results in better risk assessment and has better prognostic value^77^.

#### Application of AI in nuclear imaging to assess myocardial perfusion and CADs

An AI-based structured reporting model developed to report SPECT MPI by Garcia et al has been trained on 1,000 MPIs and has demonstrated a high percentage of agreement between AI and manual analysis. The AI-based model has achieved agreement percentages ranging from 82% to 92% for CADs, and agreement ranging from 88% to 97% for ischemia at high specificity mode^78^. Another ML-based model developed by Miller et al was trained on SPECT MPI data from 20,418 patients and was tested on another 9,019 patients. It has shown better prediction performance for abnormal perfusions than manual analysis. It has achieved 0.762 AUC, and a low Brier score (0.149) based on data collected in pre-assessments^79^. Otaki et al. designed a DL-based model that complies with SPECT MPI for the diagnosis of CAD. It was trained on data obtained from 3,023 patients and tested on 555 (total- 3,578) patients. An AUC of 0.80 per patient and 0.78 per vessel has been attained by this AI model, respectively. This has displayed better performance when compared to traditional stress total perfusion deficit^80^.

#### Application of AI in Hybrid Imaging

A hybrid model developed by Zaida et al consisted of a combination of matches from six ML algorithms, which were used to assess the survival rate and predict the risk of cardiovascular diseases (CVD). Data from 70,000 patients is obtained, and 80% is used to train AI, and the remaining 20% is used for testing. Among all the combinations, Random Forest and K-nearest neighbors (RF-KNN, Hybrid Random Forest Linear Models) had attained above 90% (92.47% to 93.08%) scores in accuracy, precision, recall, and F1-score, demonstrating their capability in assessing CVD risk^81^. Al-Issa and Alqudah have proposed a hybrid AI model based on CNN and Long Short-Term Memory (LSTM) to differentiate abnormal heart sounds caused by valvular diseases. It was trained on 1,000 audio files. This hybrid model’s performance measures show values ranging from 98.482 to 99.572 in a non-augmented data pool for accuracy, sensitivity, specificity, precision, and F1-score. In the augmented data pool, performance measures ranged from 99.864 to 99.962 for the above-mentioned parameters. AUC for the non-augmented data pool was reported to be 0.9978 and 0.9985 for augmented data, respectively^82^. Alskaf et al had developed a hybrid AI model based on multilayer perceptron (MLP) and CNN to predict the mortality rate in CAD patients based on stress perfusion CMR. A total of 1,286 patients were examined. The hybrid model has better performance when compared to individual AI models, achieving an AUC of 80% and an F1 score of 43%^83^. Table 3 summarizes the performance of AI models in cardiac magnetic resonance (CMR) and nuclear imaging.

**Table 3:** Performance of AI Models in Cardiac Magnetic Resonance (CMR) and Nuclear Imaging: Segmentation Accuracy, Diagnostic Agreement, and Prognostic Utility

Application	AI-model	Sample size	Metric Type	Performance	Ref
CMR	CNN	Trained- 814 patients Tested- 101 patients	Spearman Coefficient	ρ of RH- 0.98-0.77 ρ of LH- 0.98-0.91	^73^
CMR	CNN	Trained- 1034 patients Tested- 105 patients	Mean Dice ratio	3T- 0.93 ± 0.04 2T- 0.93 ± 0.03	^74^
LGE CMR	CNN	Trained on 144 images Tested- 152	Dice coefficients	Infarct segmentation: 64.11% MVO segmentation: 82.20%	^75^
Stress/Rest SPECT MPI	AIsR(Bayesian inference engine)	1,000 MPIs	Agreement percentage (High Specific mode)	For CAD: 82-92% For ischemia: 88-97%	^78^
SPECT MPI	ML	Trained- 20,418 Tested- 9,019	AUC Brier Score	AUC- 0.762 Brier score- 0.149	^79^
SPECT MPI	DL (CAD-DL)	Trained- 3,023 Tested- 555 Total-3,578	AUC	Per patient: 0.80. Per vessel: 0.78	^80^
CVD prediction	HeartEnssembleNet (Hybrid- RF-KNN)	70,000 80% for training 20% for testing	Accuracy- Precision- Recall- F1-score-	92.95% 93.08% 92.88% 92.47%	^81^
Phonocardiogram	Hybrid (CNN + LSTM)	1,000 audio clips	AUC	Non-augmented data: 0.9978 Augmented data: 0.9985	^82^
Stress perfusion CMR	Hybrid Neural Network (HNN- MLP + CNN)	1,286	AUC F1-score	AUC: 82% F1 score: 43%	^83^

## 6. Comparative performance, efficiency, and FDA-approved applications

### 6.1. Comparative Performance

Several studies have assessed the performance of AI versus human specialists to understand its benefit in clinical practice^9^. In one such RCT that assessed interpretation of LVEF via echocardiography, there was a lower percent change in initial and final assessment for AI interpretation (16.8%) versus sonographers (27.2%), and cardiologists could not differentiate between the two (blinding index 0.088), proving comparable results of AI for initial assessment compared to that of experienced sonographers^84^. In addition, ML models developed for diastolic dysfunction had a higher ROC value compared to the American Society of Echocardiography (ASE) 2016 diastolic guidelines grading system^9^. AI-derived algorithms such as AIEchoDx showed comparable results to those of senior cardiologists when differentiating between cardiovascular diseases, providing high AUC values for the analysis of echocardiographic videos of ASD (99.50% vs 99.26%), HCM (99.57% vs 97.21%), DCM (98.75% vs 92.75%), as well as prior MI (98.52% vs 84.20%) using diagnostic neural networks^85^. Furthermore, the use of machine learning-based echocardiography by novice nurses showed an estimated diagnostic quality of 98.8%, 92.5%, and 98.8% for LV size and function, RV size, and pericardial effusions, respectively, when blind reviewed by experienced sonographers^86^.

For CT angiography, AI outperformed radiologists in the diagnosis of coronary artery stenosis ≥50% (sensitivity: 0.92 vs 0.85, specificity: 0.87 vs 0.84, AUC: 0.96 vs 0.91) and detection of calcified plaques (sensitivity: 0.93, specificity: 0.94, AUC: 0.98)^60^. Similarly, when compared with invasive quantitative coronary angiography, it showed the highest benefit in patients with above median volume of plaque (AUC: AUC: 0.91 vs 0.77) at level 3 stenosis^87^. HeartModel AI, which uses 3D echocardiography images, showed high correlation with CMR for fully automated measures such as LVESVI (r = 0.918) and LVEDVI (r = 0.911), moderate correlation for SV (r = 0.811), and acceptable agreement for LVEF (r = 0.744)^88^.

### 6.2. Diagnostic Gains

AI can be used to standardize echocardiography images and calculate EF with a higher precision than manual methods by reducing the risk of errors and bias and allowing more reliable evaluation of LV function. Variations of these methods can also be applied to estimate diastolic dysfunction in patients with HFpEF^9,89^. Certain frame-based models (e.g., EchoNet-LVH, SE-ResNet Fusion) are under trials to differentiate major disorders causing LV thickening in early disease stages, identify subclinical conditions and disease patterns, and diagnose rare conditions that can be missed by the human eye using larger datasets and minimal segmentation of the images with limited inter-observer variability^89,90^. 3-D visualization of cardiac valves also allows accurate and rapid diagnosis of valvular heart disease, thereby improving pre-procedural planning and creating better treatment outcomes^89^. Computerized TEE also produces more accurate results in identifying thrombi within the left atrium in patients with atrial fibrillation ^9^.

Automated CAC can increase the reliability of disease prediction by accurately predicting the risk of MI and associated cardiovascular diseases^9^. Segmentation of cardiac structures enables estimation of ventricular volume, EF, and myocardial mass, which can improve pre-procedure evaluation for interventions (e.g., valve implantations)^89^. Similar algorithms can automatically find regions of fibrosis characterized by scar tissue from LGE images with reproducible assessment of cardiomyopathies and training them on larger datasets can help identify disease patterns from CMR images. A CNN with UNET-like architecture could accurately predict mortality in patients with pulmonary hypertension by measuring RVESV, EF, and mass using CMR (hazard ratio: 1.40, 0.76, and 1.15, respectively; P = .001; n = 920)^91^. In retrospective studies of nuclear imaging, XGBoost demonstrates the potential for an AI-based MACE risk prediction score, allowing patients to be appropriately assigned to stress-only imaging. In addition, the prediction of MACE using PET scanning at the 2-year follow-up has been studied, but assessment by prospective trials is required before clinical application^7^.

### 6.3. Operational Impact and Efficiency 

AI in cardiac imaging can significantly enhance operational efficiency and clinical workflow by accelerating diagnosis and optimizing utilization of resources, and evaluation of diagnostic information obtained by echocardiography and its analysis can be done much faster. More complex techniques, such as STE, have lower analysis time with high reproducibility and reduced need for expert operators when automated using CNN. Similar results for color Doppler and vector flow mapping were seen, making such techniques more accessible as part of routine clinical assessment^89^. There is an added benefit for use in emergency department settings, where such processes become more time-efficient with a lower risk of human errors^86,89^. It can also reduce high workflow by using double-checking surveillance, making echocardiography more accessible to the general population^86^.

For CAC, the reduced need for radiologist-mediated analysis of data is the main advantage. Results from the CLARIFY study demonstrated that automation of CCTA quantification allowed improved diagnostic performance for detecting stenosis with a sensitivity of 80% and a specificity of 97%. Considering that the manual estimation can take up to 30 minutes, this approach accelerated the interpretation of the data, allowing physicians to focus on more complex tasks^9,89^. Additionally, traditional methods of CMR can be lengthy and require patients to hold their breath. Deep learning models such as CNN can accelerate this scanning process while maintaining diagnostic accuracy^89^. Enhanced resolution via AI segmentation can also reduce labor requirements^9,89^.

### 6.4. Clinical AI Workflow Integration

Integrating AI-driven analysis into cardiovascular imaging promises to automate tedious tasks and accelerate diagnosis, but only if it is seamlessly incorporated into existing IT ecosystems; AI tools must “plug into” PACS/CVIS/EHR workflows without disrupting clinical operation^92^. In practice, proprietary software and data heterogeneity often complicate this, making interoperability a key challenge. Some real-world solutions demonstrate progress: for instance, Crozer-Chester Medical Center integrated AISAP’s AI-powered POCUS (CARDIO), which “seamlessly integrates with PACS and EMRs”, enabling ED physicians to perform rapid, AI-assisted bedside echo triage for chest pain^93^. Similarly, Stanford’s EchoNet-POCUS AI model achieved ~0.92 AUROC for detecting abnormal cardiac function from point-of-care echo clips in real time^94^, illustrating the feasibility of accurate AI triage using standard hardware. In January 2020, a Chinese retrospective study demonstrated an AI radiomics-based machine learning model using non-contrast CT for the detection of acute aortic syndromes with high diagnostic accuracy (AUCs up to 0.997). In contrast to CT angiography, this model is substantially faster and more readily available in emergency settings and could allow for the early triage of high-risk patients even in the absence of classical imaging signs, and might enhance clinical decision-making, resource use, and patient outcomes^95^. Despite these successes, major hurdles remain, especially workforce training: educating clinicians to use AI is widely recognized as a significant barrier^96^. Initiatives are already addressing this; for example, the SHAPE trial is testing whether medical assistants with no prior ultrasound experience can use AI guidance (Bay Labs’ EchoGPS/EchoMD) to capture diagnostic-quality echocardiograms^97^. Together, these examples underscore that rigorous IT integration standards, structured training programs, and iterative real-world pilots are essential to overcome workflow challenges and unlock AI’s potential in cardiovascular imaging.

### 6.5. Current FDA-Approved AI Tools in Cardiovascular Imaging

Several companies have developed commercially available FDA-approved AI tools for use in cardiac imaging^98^. UltraSight (FDA-approved in 2023) is an AI guidance tool for echocardiography, evaluated for its ability to assess ventricular function, valvular structures, LA and IVC sizes, and the presence of pericardial effusions. The software aims to guide novice users, allowing them to acquire images equivalent to those of expert sonographers for improved diagnostic interpretation. Images obtained using this tool allowed a concordant diagnostic interpretation in 83-96% of studies. Furthermore, quantitative analysis was possible in about 83% of these images with high correlation (r≥0.74) and minimal bias when compared to expert sonographers, which can improve point-of-care cardiac ultrasound in clinical settings^99^. Similarly, Caption Guidance (FDA-approved in February 2020), which provided real-time guidance for transducer position, was used effectively during the peak of COVID-19 and in conditions with limited resources to automatically detect EF^100^.

**Figure 1:**
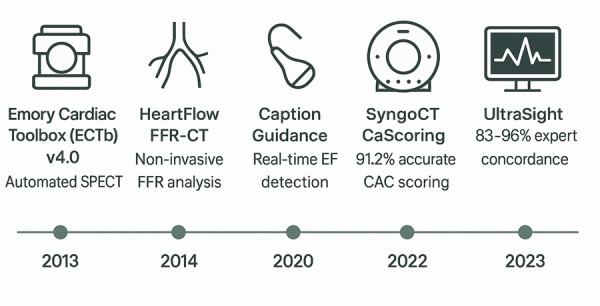
Timeline of FDA-approved AI tools in cardiovascular imaging (2013–2023). Inspired by previously published sources and data in References ^78^, ^99^, ^100^, ^101^, and ^102^.

Applications in CT include SyngoCT CaScoring VB60 (FDA 501(k) clearance in May 2022), developed for automated CACS. It was tested in a retrospective trial in patients undergoing routine CACS by CT, where it performed with a high accuracy of 91.2% and saved about 60% of time when compared to manual scoring^101^. HeartFlow’s FFR-CT (FDA-approved in November 2014) was assessed in patients from the ADVANCE registry, where it provided improved diagnostic and prognostic value over anatomical assessment done alone at the 1-year mark post CTA, with consistent outcomes regardless of age^102^. Meanwhile, the FDA-approved tool, Syngo.Via has also been utilized to assess and compare CMR images to echocardiography, and it avoided overestimation of LVEF by human operators^103^. Emory Cardiac Toolbox (ECTb) Version 4.0 (FDA-approved in February 2013) was used to automatically generate reports from SPECT images, where it successfully detected CAD and ischemia comparable to experts (p: 5.33, p: 5.37, respectively)^78^**.** Figure 1 illustrates the timeline of FDA-approved AI tools in cardiovascular imaging.

### 6.6. Economic Considerations

Integration of AI Tools in cardiac imaging requires significant initial investments and ongoing maintenance costs. Its main advantage lies in improving long-term economic benefits since it can save costs by reducing unnecessary repeat imaging and lowering workload on operators. Furthermore, improved efficiency and a reduction in the required trained manual labor can minimize operational expenses. These advantages improve patient outcomes, resulting in shorter durations of hospital stays and timely diagnoses^9,89,104^. In conclusion, there is significant potential for transforming healthcare delivery, optimizing resource allocation, and improving patient care by enhancing diagnostic capabilities and operational workflows.

## 7. Challenges, Limitations & Ethics

### 7.1 Bias in Healthcare Imaging

Bias in imaging techniques can manifest as social, cognitive, or statistical. A lack of diversity in data for the training models further amplifies existing sociocultural bias, causing suboptimal outcomes for specific groups within the population. Cognitive bias refers to a deviation of the system from objective judgment, leading to diagnostic errors. Statistically, differences between the expected and true values can be implicated as systematic errors (i.e., flawed models with output unrepresentative of real conditions) in contrast to random errors occurring due to unpredictable changes or chance (i.e., noise)^105,106^. In a study evaluating DL models for automatic segmentation of the myocardium and ventricles from CMR images in a diverse population, there were differences in the DSC values of each racial group: 92.39 ± 1.86 for whites and lower for all other races (Mixed: 84.49 ± 2.08, Asians: 88.29 ± 2.43, Black: 86.02 ± 1.79, Chinese: 83.77 ± 2.32, and others: 85.96 ± 3.81). There was no difference between males (91.35 ± 3.12) and females (91.32 ± 3.29). Strategies to lessen racial bias include ensuring racial balance during training, integrating an AI race classifier to capture racial variations, and training separate models for each racial group^107^. Addressing this bias in imaging using diverse data and targeted models ensures fair and accurate outcomes across all populations.

### 7.2 Black Box in Deep Learning

Deep neural networks originate from complex statistical models with multiple parameters, resulting in non-transparent internal decision-making referred to as the “black box” problem. This limits their credibility in critical healthcare applications and necessitates explainable artificial intelligence (XAI) that adheres to three main principles: transparency, interpretation, and explanation^108,109^. A scalable approach based on a transition matrix based on clinical guidelines (to simplify and map the datasets) can enhance the interpretability in processing medical images by converting them into user-friendly features for healthcare professionals. This method was tested on CMR datasets for classification of heart disease with strong agreement to expert evaluations (Cohen’s Kappa coefficient: 0.80). Such results demonstrate the reliability of this solution in providing understandable, accurate, and justifiable explanations of the decisions made by the DL model, suggesting benefit in application across broader medical domains^108^.

### 7.3 Privacy Concerns

All AI-based techniques usually require access to large amounts of health-related and other personal data, either from prior health records or directly from patients, and the use of this sensitive information can reduce their willingness to trust such models over concerns of privacy and data protection^110^. Global initiatives developed to address this issue include (1) HIPAA (est. 1996), enacted as a federal law to create national standards to handle health information, prohibiting its disclosure without the patient’s knowledge or consent; (2) GDPR (est. 2018), applicable throughout the EU, promoting the creation of digital systems that protect the user’s privacy by imposing strict rules over organizations (within or outside the EU) with access to data of EU citizens; and (3) the Global Initiative on Ethics of Autonomous and Intelligent Systems (launched in 2016 by IEEE), which formulates standards to make AI more secure and ethical for society^110^. However, there is an additional concern of cross-continent data sharing, because the differences in the laws governing health information can create loopholes, resulting in the exploitation of data^110^. Other solutions are AI-based model protection like federated learning (where multiple devices collaborate to train a model while maintaining confidentiality of input), differential privacy (addition of noise or randomness to conceal an individual's information within the dataset), cryptographic techniques (encryption of data before training and testing), and hybrid techniques that combine all the mentioned methods. Therefore, the responsible adoption of AI in healthcare is reliant on more comprehensive data governance and integrating privacy-preserving methodologies to maintain patient trust and protect sensitive health information^110,111^.

### 7.4 Ethical Considerations 

Ethical aspects ensure that new technology follows the six principles set by the World Health Organization (WHO) for the application of AI in healthcare: (1) autonomy, (2) beneficence, (3) explainability, transparency, and intelligibility, (4) accountability, (5) equity and inclusivity, (6) sustainability and responsivity^112^. Although these principles address bias to ensure that all approved AI tools prioritize human well-being and public safety, meticulous testing and validation are necessary to minimize harm. The lack of diversity among developers and testing data further propagates this issue; hence, unconscious bias testing is important in the development phase. Another major issue is privacy, transparency, and informed consent; patients should be given complete and comprehensive details of how AI will be used in their care and how it may affect their management (including limitations). This cannot always be executed since such tools are deeply integrated into healthcare, and an alternate would be to mandate human oversight (HITL) and ensure rigorous monitoring of the system^112,113^. In addition, Article 6 of the European Union’s AI Act serves as a legal framework to regulate AI use for high-risk applications (e.g., healthcare), and Article 10 mandates relevant training and validation before intended use. This act also requires strict data governance to reduce negative impact or discrimination for the patients^112^. Implementing these regulations would ensure patient safety, trust, and equity.

### 7.5 Liability Risks 

Liability in case of errors or misdiagnosis is a major problem with the use of AI, for which there is no definitive answer yet^114^. Negligence may arise from a failure during the programming stage, in the supervision by a healthcare expert, or even from the algorithm itself^114,115^. When involving DL, this problem is further perpetuated due to the “black box” issue, creating uncertainty for both the manufacturers and the supervising physician. The legal handling can be simplified by considering the degree of autonomy of the AI tool; when an autonomous tool is used correctly and independently, liability falls on the developer, whereas for assistive tools, clinicians are fully capable of independent assessment and will be held liable regardless of AI-generated errors. Vicarious liability can be applied if we consider the AI working as a subordinate in the facility, “respondeat superior,” or negligence of the assistant is attributed to the supervisor (in this case, the clinician or healthcare facility). On the other hand, product liability requires a defect in the product and considers the manufacturer responsible. However, there is a major concern with the self-evolving nature of these algorithms, and often the algorithm causing harm is different from the original, so the customizing facility can also be held responsible (enterprise liability). In summary, a common and strict liability approach with a more distributed conception would be the best solution to manage errors^115^**. **

### 7.6 Regulatory Approval

The current regulation of AI/ML tools is managed at the global level by the US Food and Drug Administration (FDA) for evaluating safety and effectiveness. In the EU, passage of the Medical Device Regulation (MDR) and the In Vitro Diagnostic Regulation (IVDR) in 2017 was an innovative revision to the previous regulatory framework^116,117^. The regulatory process requires convergence of both patient and industry perspectives. From a patient's perspective, a regulatory framework should safeguard not only their safety but also their consent as direct participants. According to industry perspectives, aligning rules helps to prevent overlap between general and health-care-specific regulations. On the other hand, the regulators respond to concerns from various groups—the general society, patients, industry, and healthcare providers. The major challenge is establishing standard criteria for the clinical validity of AI^116^. In addition, gaps in current regulation include overlap of task and algorithm, superficial definitions of diagnostic tasks, lack of comparability, insufficient characterization of performance and safety elements, limited resources to validate performance at installation sites, and inherent conflicts of interest^118,119^. All stakeholders must participate in creating a risk-based and resilient approach, with continuous surveillance and clinical evaluation, ensuring high-quality data feeding with governance and data control to account for error, inappropriate use, and real-world shifts in data to allow for improved regulation of these tools^119^.

In summary, the application of AI/ML tools in cardiac imaging techniques to enhance workflow and efficiency can also present several challenges: lack of transparency in decision-making, bias towards specific subsets of the population, privacy and data safety, ethical and legal risks, and finally, insufficient standards of regulation. These issues can be addressed through an adaptive interdisciplinary approach to balance their benefits with well-rounded regulation to ensure they focus on developing patient welfare in healthcare.

## 8. Future Directions

### 8.1 Emerging trends in cardiovascular imaging

Healthcare is becoming more personalized and precise due to the development of AI technologies and their integration into various fields of medicine, such as cardiovascular imaging. This will enhance patient outcomes by providing early diagnosis and prompt management of CVD^120^.

Although AI models have numerous advantages, some limitations prevent them from being widely used in medicine. One of them is that most of the AI models are trained on data that is small, narrow, and not diverse. This could result in lower generalizability and over-fitting. To overcome this issue, Federated Learning (FL) or Transfer Learning methods can be used, which train the models with data from different sites without centralising it. This leads to a decreased risk of data leaks or unauthorized access as the data is not sent over to other networks^121,122^. Due to training with multiple datasets, the model becomes familiar with rare cases, which increases its sensitivity and results in decisions that are less biased. The performance of such models is similar to those trained on centrally hosted datasets and much better when compared to models trained with data from a single institution, according to the latest studies^123^.

Another disadvantage of AI models is their “unexplainable” feature, also known as “black-boxes”. This means that how the AI reaches a specific conclusion or diagnosis is not known. The lack of transparency raises questions regarding its working^124,125^. Explainable AI methods are being developed to resolve this issue^126^. Class Activation Mapping (CAM) is a technique that comes under Explainable AI. CAM can identify the input patterns within the deep neural network, which leads to the activation of certain outputs and shows the findings on the image. This helps the physicians who use AI to better understand the reasoning of the model and make informed decisions^127^.

AI can also serve as a means of bringing together different imaging modalities, which can be extremely helpful, especially in the diagnosis of heterogeneous diseases, for example, heart failure and atrial fibrillation^128^. The multimodality AI approach combines information, in the form of image, text, audio, video, and language, obtained from different imaging methods. Each modality offers important additional data, resulting in accurate outputs. A study was done to distinguish between the causes of left ventricular hypertrophy by merging data from ECG and echocardiography. The multimodal AI was found to have higher sensitivity and specificity compared to physicians^129^.

Therefore, as AI technologies are evolving rapidly, it is changing how cardiovascular imaging is used, by improving image quality, reducing the time and workforce required for image analysis, and providing prognostic information^10,130^.

### 8.2 Wearable AI Devices

ECG is an essential and most frequently used non-invasive test in cardiology. However, to record and interpret an ECG, the individual is required to visit a healthcare professional. This results in paroxysmal arrhythmias being undetected. The integration of ECG monitoring and AI-based ECG interpretation into wearable devices like smartwatches has led to widespread access to ECG^131^. Furthermore, there has been an increase in the detection of arrhythmias^131,132^. In case of AF, early detection is crucial to avoid progression and development of complications such as stroke. As most cases have either nonspecific symptoms or are asymptomatic, patients are unaware of their condition and hence do not seek medical care. Using wearable devices with AI technology, early and timely detection is possible, resulting in improved treatment outcomes^133^.

Apart from arrhythmias, such devices can also be helpful in the monitoring of patients with other CVDs, for example, CHD, Left Ventricular Systolic Dysfunction (LVSD) with fewer leads, and Myocardial Infarction (MI). In patients with CHD, wearable devices with ECG can identify those at risk for fatal outcomes. This allows patients with medium or high risk to seek further medical attention and preventive treatment^133^. Previous studies have shown that a smartwatch AI-based ECG model for the identification of LVSD had an AUROC of 0.93, regardless of the type of device used (eg, Apple Watch, Samsung Galaxy Watch). In addition, an AUROC of 0.85 was attained by a model designed to identify MI using a smartwatch 6-lead ECG^134^.

Despite having multiple advantages, wearable AI devices have their fair share of limitations. Integration of the wearable data into the Electronic Health Record (EHR) is not easy. As the data is recorded outside the hospital setting, it may not be accurate, reliable, and raise privacy concerns. In addition to this, as an increased number of individuals with non-urgent cases or false-positives visit physicians, this can lead to missing out on individuals with serious problems. Therefore, further research regarding the clinical significance of such AI technologies is required before they are widely incorporated into clinical practice^133^.

### 8.3 Real-Time AI

Apart from reducing workload by minimising the diagnosis time, AI can also help segregate urgent cases from non-urgent ones and thus reduce the burden on the physicians.

For example, one of the most common reasons for ER visits is chest pain, which in more than 50% cases is non-cardiac in origin and non-serious. Differentiating between cardiac and non-cardiac chest pain is quite difficult. Although conventional diagnostic tests, such as highly sensitive troponin levels, can report if the cause of chest pain is due to MI, they require time and are unsuitable in urgent scenarios. Therefore, AI tools are needed that can rapidly interpret biomarkers and various datasets and help in quick decision-making. A study was performed using an AI tool called RAPIDx AI. Among ER registrars and advanced trainees, the tool achieved optimal comprehension and preference scores and helped in immediate decision making^135^.

Furthermore, in case of Occlusion Myocardial Infarction (OMI), percutaneous coronary intervention (PCI) is required without delay. Due to this, the emergency medical services (EMS) personnel immediately inform the catheterization labs. But not all cases are OMI, and this leads to an increased burden on the labs. To overcome this problem, AI algorithms are being developed with improved ability to interpret ECGs. According to a study, compared to the judgment of EMS professionals, an AI-based tool had a lower OMI false positive rate^136^.

Therefore, AI-based tools can improve the prognosis of the patient by making accurate yet timely diagnoses and simultaneously lowering the load on the healthcare system.

### 8.4 AI in Resource-Limited Areas

Not only in resource-rich areas, but in resource-limited areas of the world as well, the use of AI and digital health in medicine is becoming increasingly popular^137^. Especially in resource-limited regions and countries, AI can be of tremendous help as there is a shortage of experienced and trained radiologists. Additionally, it can also be beneficial in the absence of state-of-the-art medical infrastructure^138,139^. One of the most important advantages that AI tools offer in such areas is that they significantly reduce the cost of diagnostic tests since they do not require knowledge from highly skilled physicians and expensive machinery^140^. Therefore, the use of AI ensures that high-quality medical care is delivered.

### 8.5 Challenges and Legal Liabilities

Although AI offers numerous advantages in the field of cardiac imaging, there are also some challenges that it faces. The most important one is the type of dataset the AI model is trained on. The dataset should be diverse in order to attain good generalisability and avoid bias^135,136^. Furthermore, medical professionals and patients often misunderstand AI and do not trust it. Adding human judgment in AI-based tasks can help resolve this issue^141^.

Apart from these challenges, there are also legal issues that should be taken into consideration. A question which frequently arises is whether the physician will be responsible if the AI model commits an error, as the treatment and care of the patient is the duty of the physician^142^. Therefore, these hurdles will have to be overcome before AI is completely integrated into the healthcare system.

## OPEN QUESTIONS

● Generalizability to the population as a whole: How can federated and transfer learning be utilized to achieve robust performance for individuals in a range of ethnic, age, and comorbid disease groups without compromising data sharing^121-123^?

● Trade-off between explainability and performance: How much transparency of the model (XAI techniques, CAM maps) is needed for clinician trust and regulatory agency approval, and what implications does this have for diagnostic accuracy and clinical implementation?

● Integrating into the clinical workflow: What are the optimal AI-human interaction paradigms (e.g., real-time triage, HITL oversight, automated reporting) to maximize efficiency gains without trading off safety in high-acuity settings?

● Regulation and liability: What processes should be used to “oversee” dynamic “learning” algorithms in operation, to handle degradation, to ensure patient safety, and to apportion liability among the developers, organisations, and practitioners?

● Cost-effectiveness and access: What will be the sustained economic implications of AI implementation on health-system costs, and how can AI solutions be scaled to resource-constrained settings to mitigate inequalities?

## 9. Conclusion

AI has dramatically changed the field of cardiovascular imaging, leading to substantial gains in clinical decision-making across the spectrum of capabilities, operations, and diagnostic confidence. The review focuses on the major developments, including application of convolutional neural networks (CNNs) in automated coronary artery calcium (CAC) scoring with near-perfect agreement to manual scoring, high-sensitivity plaque detection in coronary computed tomography angiographic images, and near real-time interpretation of echocardiographic images using ML applications, such as EchoNet-Dynamic. Standardization of image acquisition, reduction of inter-observer variability, and the acceleration of workflow in urgent scenarios have all been helped a lot with these advances.

Moreover, AI-enabled tools have already demonstrated clinical usefulness and scalability in cardiac MRI and nuclear imaging by automating tissue characterization, volumetric quantification, and even viability detection. FDA-approved tools, including Aidoc, HeartFlow, and Caption Health in particular, signal a movement from experimental models to regulatory-validated solutions with demonstrated benefits to patients.

However, there are still significant unresolved issues despite these advances. It is not generalizable as most of the models are trained on homogeneous datasets. The explainability of AI decisions continues to limit clinical trust. There are, however, a number of practical barriers that make real-world deployment difficult, such as reimbursement confusion, lack of interoperability, and ethical concerns about data ownership, bias, and liability. In addition, there is also a scarcity of current models that include detailed patient-specific risk factors or multimodality data.

Wearable devices, multimodal AI, and federated learning may make cardiovascular imaging a predictive and preventive platform in the future. AI systems that can diagnose disease and deliver individualized treatment decisions in real time represent a major advancement. But it will require extensive validation, open algorithm design, and close coordination between regulators, data scientists, and clinicians.

In summary, while AI has made tremendous headway in cardiovascular imaging, solving the current challenges will shape its future impact. AI has the potential to not only improve but also completely transform the way cardiovascular diseases are identified, tracked, and managed with thorough research, practical testing, and ethical implementation.

## Bullet Points

● *AI-based echocardiography can provide human expert-level accuracy of image categorization, automated LVEF and GLS measurement, and disease detection (e.g. MR, AS) with ≥99% sensitivity/specificity, along with a nearly dramatic reduction in inter-observer variability^28-32,37-40^.*

● *Deep-learning based CAC scoring on gated and non-gated CT demonstrates near-perfect agreement with manual Agatston scoring of (κ=0.89; r=0.92) on study populations of 79 to 1,811 scans, allowing for rapid, reproducible atherosclerosis quantification without additional radiation^50-53^.*

● *Automated plaque quantification and stenosis detection on CCTA (308–894 scans) achieve ICC≥0.84 for plaque volumes and 96% ACC for ≥50% stenosis, and are equivalent to IVUS and expert readings; and they reduce the analysis time from tens of minutes to seconds^55,58-61^.*

● *AI models for CMR segmentation and perfusion (814–1,034 patients) provide Dice coefficients ≥0.93 and Spearman ρ ≥0.91 for chamber volumes and robust infarct/MVO detection (Dice 64–82%), faster workflow, and scalable strain and tissue characterization^73-75^.*
